# The Role of NRF2 in Mycobacterial Infection

**DOI:** 10.3390/antiox10121861

**Published:** 2021-11-23

**Authors:** Masashi Matsuyama, Mizu Nonaka, Masayuki Nakajima, Yuko Morishima, Yukio Ishii, Nobuyuki Hizawa

**Affiliations:** Department of Respiratory Medicine, Faculty of Medicine, University of Tsukuba, Tsukuba 305-8575, Ibaraki, Japan; mizu.nonaka85@gmail.com (M.N.); m.nakajima12@gmail.com (M.N.); mk01a231@md.tsukuba.ac.jp (Y.M.); ishii-y@md.tsukuba.ac.jp (Y.I.); nhizawa@md.tsukuba.ac.jp (N.H.)

**Keywords:** ROS, NRF2, ARE, TB, NTM, IFN-γ, HO-1, NRAMP1

## Abstract

The incidence of pulmonary nontuberculous mycobacterial (NTM) infection is increasing worldwide, and its clinical outcomes with current chemotherapies are unsatisfactory. The incidence of tuberculosis (TB) is still high in Africa, and the existence of drug-resistant tuberculosis is also an important issue for treatment. To discover and develop new efficacious anti-mycobacterial treatments, it is important to understand the host-defense mechanisms against mycobacterial infection. Nuclear erythroid 2 p45-related factor-2 (NRF2) is known to be a major regulator of various antioxidant response element (ARE)-driven cytoprotective gene expressions, and its protective role has been demonstrated in infections. However, there are not many papers or reviews regarding the role of NRF2 in mycobacterial infectious disease. Therefore, this review focuses on the role of NRF2 in the pathogenesis of *Mycobacterium tuberculosis* and *Mycobacterium avium* infection.

## 1. Introduction

The incidence of pulmonary nontuberculous mycobacterial (NTM) infection is increasing worldwide [1,2], and its clinical outcomes with current chemotherapies are unsatisfactory. The incidence of tuberculosis (TB) is still high in Africa, and the existence of drug-resistant TB is also an important issue for treatment [3,4]. To discover and develop new efficacious anti-mycobacterial treatments, it is important to understand the host-defense mechanisms against mycobacterial infection. During infection with microorganisms, phagocytic cells produce an excess of oxidants that contribute to the clearance of pathogens, but they sometimes cause tissue injury [5]. Therefore, the regulation of oxidative stress may be one of the important host-defense mechanisms during infection. Nuclear erythroid 2 p45-related factor-2 (NRF2) is a redox-sensitive transcription factor that plays an important role in the antioxidant and detoxification activities of the body [6]. Although there are some papers regarding the role of NRF2 in mycobacterial infectious disease, not enough review articles have focused on it. In this review, the epidemiology of *Mycobacterium tuberculosis* and pulmonary NTM disease is examined first. Second, our understanding of oxidative stress and reactive oxygen species in mycobacterial infection is discussed. Third, the detailed role of NRF2 in tuberculous infection and nontuberculous mycobacterial infection is presented. Fourth, the relationships between aging and NRF2 in terms of mycobacterial infection are mentioned. Last, the possibilities of developing new therapeutic options targeting NRF2 and oxidative stress for NTM and TB infections are discussed.

## 2. Epidemiology of *M. tuberculosis* and Pulmonary NTM Disease

Although the global incidence of TB peaked around 2003 and appears to be decreasing slowly [7], it is estimated that more than 1.7 billion people are infected with *M. tuberculosis* [7,8]. According to the global tuberculosis report 2020 from the World Health Organization, in 2019, 10 million individuals became ill with TB. In 2019, 1.2 million human immunodeficiency virus (HIV)-negative people died of TB, and there were an additional 208,000 deaths among HIV-positive people [7]. In addition, resistance to commonly used anti-tuberculous drugs is increasing. Multidrug-resistant TB (MDR-TB) occurs worldwide; the highest numbers of estimated MDR-TB cases are found in China, India, Russia, and the countries of the former Soviet Union [3,9,10,11]. In MDR-TB patients who received treatment, overall outcomes were poor; only about 56% of patients were considered cured. As improved diagnostic testing becomes available, MDR-TB will likely be recognized as the major threat to global TB control over the next several decades.

On the other hand, the incidence and prevalence of pulmonary NTM infection have increased worldwide in recent decades [1,2]. There are over 190 species and subspecies of nontuberculous mycobacteria, some of which can cause disease in humans and can affect pulmonary and other body sites [12]. As for pulmonary NTM infection in adults (without cystic fibrosis or HIV infection), the most common NTM pathogens are *Mycobacterium avium* and *Mycobacterium intracellulare*, the so-called *Mycobacterium avium-intracellulare* complex (MAC), among the slowly growing nontuberculous mycobacteria, and *Mycobacterium abscessus* among the rapidly growing nontuberculous mycobacteria. Although some may say improved diagnostic methods, increased physician awareness, and global warming might have led to such an increase in pulmonary NTM infection, the detailed reasons are still unknown. Nevertheless, innovative advances in terms of pathophysiology and diagnostic and treatment methods are needed to overcome the increase in pulmonary NTM disease. In addition, unlike drug-susceptible TB infections, no drug has yet been developed that can cure NTM infections completely.

Therefore, it is very important to understand the pathophysiology of mycobacterial infection in order to develop new efficacious anti-mycobacterial treatments and to control the refractory diseases, such as MDR-TB and MAC diseases. In this review, the focus is on the role of NRF2 in the pathogenesis in *M. tuberculosis* and *M. avium* infections.

## 3. The Effects of Reactive Oxygen Species in General Bacteria and Mycobacteria

**Figure 1:**
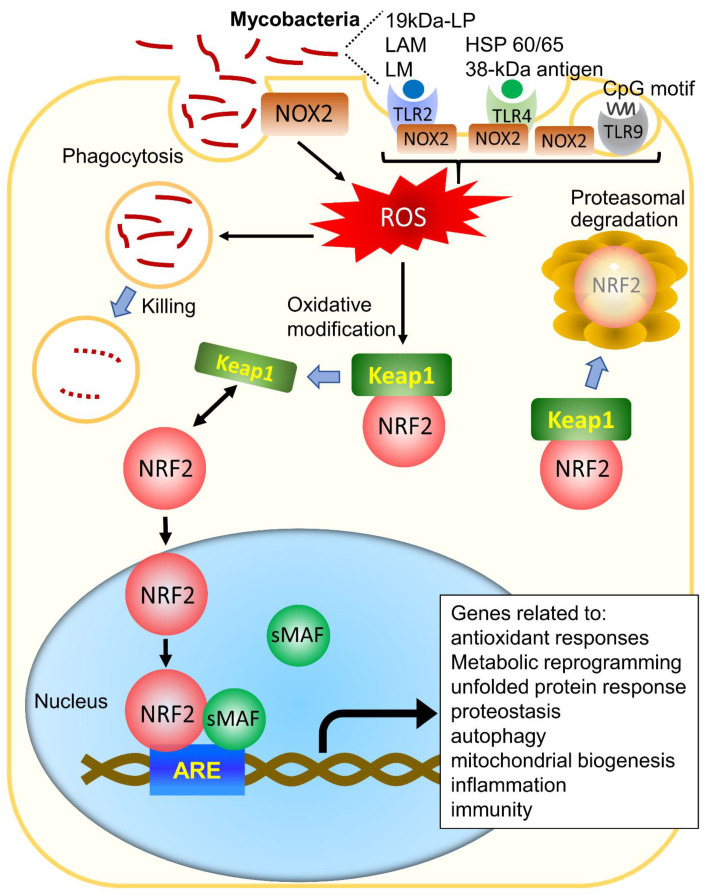
**Activation of NRF2 in mycobacterial infection.** Mycobacteria are phagocytosed by phagocytes and induce ROS via NOX2 on the phagosomal membrane. Several mycobacterial-derived components also induce ROS via TLR-mediated NOX2 activation. The induced ROS kills mycobacteria in the phagocytes. On the other hand, under exposure to ROS, NRF2 dissociates from Keap1, and NRF2 travels to the nucleus, where it induces not only genes related to antioxidant responses but also genes related to metabolic reprogramming, unfolded protein responses, autophagy, proteostasis, mitochondrial biogenesis, immunity, and inflammation, under the regulatory influence of ARE. Abbreviations: ARE, antioxidant response elements; TLR, Toll-like receptor; NOX2, nicotinamide adenine dinucleotide phosphate (NADPH) oxidase 2; ROS, reactive oxygen species; KEAP1, Kelch-like ECH-associated protein-1; NRF2, nuclear erythroid 2 p45-related factor; sMAF, small musculoaponeurotic fibrosarcoma; 19kDa-LP, 19kDa-lipoprotein; LAM, lipoarabinomannan; LM, lipomannan; HSP, heat shock protein.

When microorganisms invade the body, they are recognized, engulfed, and phagocytized by inflammatory cells, such as macrophages, neutrophils, and dendritic cells. Pathogens induce the expression of nicotinamide adenine dinucleotide phosphate (NADPH) oxidase (NOX), particularly NOX2. Then, the nitric oxide synthase in phagocytic cells leads to increased production of reactive oxygen species (ROS) and reactive nitrogen species (RNS) [5,13,14,15,16] (Figure 1). This process, which is crucial for pathogen clearance, occurs in many infections.

Macrophages express pattern-recognition receptors, such as Toll-like receptors (TLRs) and C-type lectins, which recognize particular bacterial products known as pathogen-associated molecular patterns [17,18,19]. TLR1, TLR2, and TLR6 are expressed on the plasma membrane and recognize mycobacterial lipoproteins, proteins, and glycolipids, whereas TLR3, TLR7, and TLR8, which are present intracellularly, traffic from the endoplasmic reticulum to endolysosomal compartments, where they detect mycobacterial nucleic acids [20]. TLR activation induces the generation of ROS by NOX2 for intracellular mycobacterial killing [21,22] (Figure 1).

Oxidative stress is induced by the consequence of an imbalance between oxidants and antioxidants in cells. Free radicals, including ROS and RNS, are highly unstable and react with biological macromolecules, thereby inflicting damage to DNA, proteins, and lipids. Oxidants activate the redox-sensitive NF-κB signaling pathway, which induces the expression of cell adhesion receptors, chemokines, and proinflammatory cytokines, which are associated with the production of free radicals and persistence of inflammation [5]. The role of oxidative stress in infection is only partially understood. Basically, oxidative stress has long been shown to inhibit the growth of bacterial organisms. However, some intracellular bacteria, including *M. tuberculosis*, *M. abscessus*, and *M. avium*, grow rather well in oxidizing environments. Actually, *M. tuberculosis* is more resistant to ROS than general bacteria and other intracellular parasites, including Listeria and Salmonella [23,24], because *M. tuberculosis* has some antioxidant enzymes, such as superoxide dismutase (SOD), alkyl hydroperoxide reductases (AhpC), and catalase peroxidases, to adapt to oxidative stress [25]. The *secA2* gene encodes a protein required for the secretion of SOD A. The depletion of this gene leads to enhanced ROS production, apoptosis, and loss of virulence [26]. Thus, *M. tuberculosis* prevents apoptosis of host cells to reduce ROS levels by producing antioxidant enzymes such as SOD A.

*M. abscessus* is a rapidly growing mycobacterium, and infections with *M. abscessus* have also been increasing globally. Experiments on THP-1-derived macrophages showed that growth of *M. abscessus* was rather enhanced in the presence of oxygen free radicals, but it was suppressed in the presence of an ROS scavenger, Mn (III) tetrakis (N-ethylpyridinium-2-yl) porphyrin (MnTE-2-PyP) [27]. In addition, Kim et al. reported that the rough type of *M. abscessus*, but not the smooth type of *M. abscessus*, enhanced type 1 interferon (IFN-I) secretion via bacterial phagosomal escape, contributing to increased virulence [28]. Moreover, recently, they showed that mitochondrial ROS-dependent IFN-I production inhibits IL-1β production, further contributing to the intracellular survival of the rough type of *M. abscessus* in murine macrophages [29].

*M. avium* also has a way to combat oxidative stress [30,31]. *M. avium*-infected macrophages produce and release large amounts of superoxide anions, and Cu-Zn SOD was characterized as a strategy by *M. avium* to avoid such host defense mechanisms [30]. McNamara et al. also reported that AhpC was expressed in *M. avium*-infected macrophages [31]. AhpC is an antioxidant enzyme that may be an essential factor in intracellular survival for both *M. tuberculosis* and *M. avium* [26,31]. Li et al. identified a number of mutations in *M. avium* genes of unknown function that are associated with attenuation of *M. avium* bacterial burdens in mice. In addition, some of the genes were associated with the reduction of oxidative stress [32]. Thus, in addition to SOD and AhpC, *M. avium* may have several genes related to resistance to ROS and RNS [32].

**Figure 2:**
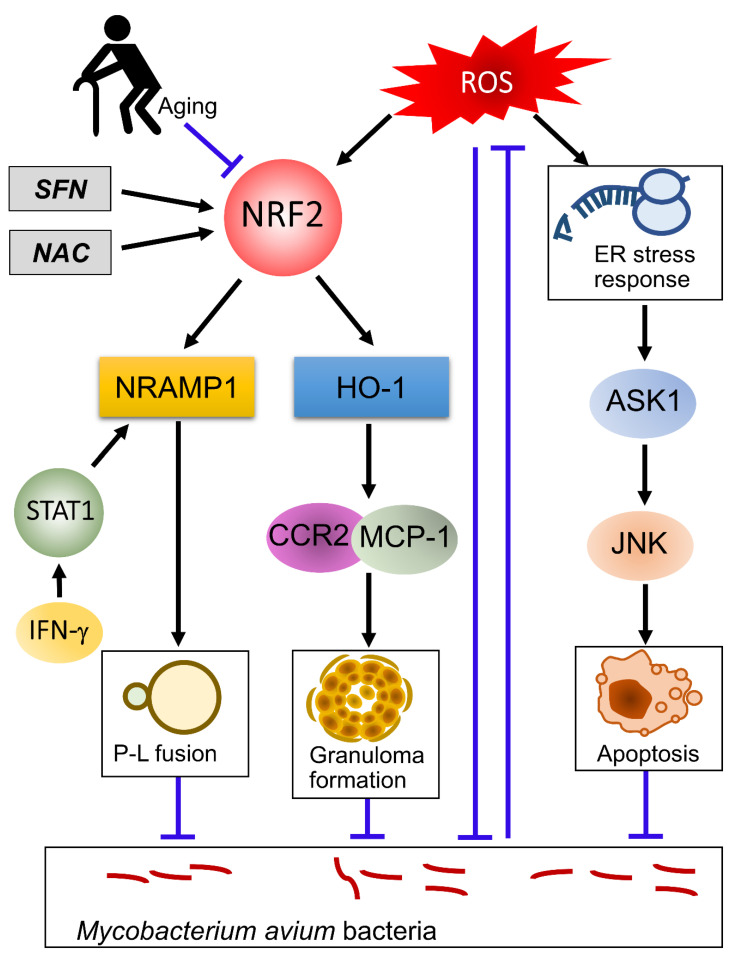
**Putative schematic diagram of the role of NRF2 in NTM infection.***Mycobacterium avium* (*M. avium*) infection induces ROS and activates NRF2, which induces HO-1 and NRAMP1 and promotes granuloma formation and P-L fusion. NRAMP1 is also induced by IFN-γ/STAT1 signaling pathway during mycobacterial infection [44]. ROS not only kills *M. avium* directly, but also induces apoptosis of infected cells. On the other hand, *M. avium* has a way to counteract ROS. NRF2 is activated by NAC and SFN. It has also been reported that aging suppresses the function of NRF2. Abbreviations: ROS, reactive oxygen species; NRF2, nuclear erythroid 2 p45-related factor; NRAMP1, natural resistance-associated macrophage protein 1; P-L fusion, phagolysosome fusion; HO-1, heme oxygenase 1; SFN, sulforaphane; NAC, N-acetyl cysteine; ASK1, apoptosis signal-regulating kinase 1; JNK, c-Jun N-terminal kinase; ER, endoplasmic reticulum; TLR, Toll-like receptor; MCP-1, monocyte chemotactic protein-1; CCR2, chemokine receptor 2, IFN-γ, interferon-gamma; STAT1, signal transducers and activators of transcription.

On the other hand, ROS act as intracellular signaling molecules [33]. The purified protein derived from *M. tuberculosis* activates TLR-2-dependent ROS production [34]. When macrophages are infected with *M. tuberculosis*, ROS cause proinflammatory responses by activation of the apoptosis signal-regulating kinase, which is a member of the mitogen-activated protein kinase kinase kinase (MAP3K) family that activates the Jun N-terminal kinase and the p38 pathways [35,36]. Phagosomal ROS, produced after *M. tuberculosis* infection through induction of NOX2, are necessary for stimulating the production of TNF, which is essential for the induction of apoptosis and anti-mycobacterial defenses [37]. As for *M. avium* infections, it has also been reported that TLR-mediated induction of ROS induces apoptosis through mitochondrial or ER pathways [38,39,40]. This induction of apoptosis is involved in the suppression of the intracellular survival of *M. avium* in macrophages (Figure 2). These results suggest that ROS induce apoptosis of mycobacteria-infected macrophages. In some mycobacteria, such as *M. tuberculosis*, *M. abscessus*, and *M. avium*, induction of antioxidant molecules is necessary for the prevention of ROS-mediated apoptosis of host cells. In addition to dealing with ROS, *M. tuberculosis*, *M. abscessus*, and *M. avium* can escape from bactericidal and apoptotic processes in macrophages by various mechanisms [41,42,43]. It is therefore difficult to kill mycobacteria by macrophages alone. For efficacious elimination of mycobacteria, the interaction of infected macrophages and T-cells, especially Th1 cells, is required; IL-12, produced by infected macrophages, differentiates naïve T cells from Th1 cells and activates them to produce IFN-γ. The IFN-γ secreted by these cells induces mycobactericidal activity of infected macrophages by amplifying ROS production [44]. This control mechanism of mycobacterial infection is called the IFN-γ/IL-12 axis [45,46,47].

## 4. The Role of NRF2 in the Regulation of Oxidative Stress and Its Role Other than the Antioxidant Effect

NRF2 is a transcription factor that regulates the cellular defense against toxic and oxidative insults through the expression of genes involved in the oxidative stress response and drug detoxification. NRF2 activation makes cells resistant to chemical carcinogens and inflammatory challenges. The mechanisms of NRF2 activation by oxidative stress have been understood at a molecular level. Under non-oxidative stress in normal cells, NRF2 is kept in the cytoplasm by binding with Kelch-like ECH-associated protein-1 (KEAP1), a cellular stress sensor protein, and undergoes proteasomal degradation by KEAP1-dependent ubiquitination [48] (Figure 1). In the presence of reactive oxygen species (ROS), KEAP1-dependent ubiquitin ligase activity is suppressed, and NRF2 can move into the nucleus. In the nucleus, NRF2 forms a heterodimer with small musculoaponeurotic fibrosarcoma (MAF) proteins and binds to the antioxidant response element (ARE) consensus sequence [49] (Figure 1).

Interestingly, in addition to antioxidant responses, NRF2 is associated with many other cellular processes. Türei et al. developed an online resource, NRF2-ome, to provide an integrated and systems-level database for NRF2 [50]. The database contains 7777 proteins and 35,967 interactions [50]. Among the 7777 proteins, 227 that interact directly with NRF245 are transcription factors (TFs) directly regulating NRF2, whereas 165 TFs regulate miRNAs capable of downregulating NRF2. Moreover, 7252 proteins in the NRF2-ome database are encoded by genes regulated by NRF2. These findings suggest that NRF2 activity is regulated through a complex transcriptional and post-translational network that makes it orchestrate the cell’s response and adaptation to various pathological stressors for homeostasis maintenance. In fact, previous reports showed that NRF2 exists at the center of a complex regulatory network and establishes NRF2 as a truly pleiotropic transcription factor, related to metabolic reprogramming, unfolded protein responses, autophagy, proteostasis, mitochondrial biogenesis, immunity, and inflammation [51,52,53,54,55,56,57,58,59] (Figure 1).

## 5. The Role of NRF2 in Tuberculous Infection

TB patients show high levels of oxidative stress markers and depletion of antioxidants, including vitamin C, vitamin E, and glutathione [60]. A previous study of a TB guinea pig model showed the presence of oxidative stress markers and increased oxidant-mediated lung and spleen lesions. The expression level of NRF2 in *M. tuberculosis*-infected cells was increased, but NRF2 was mainly localized in the cytoplasm. These findings suggest that NRF2 is not fully activated in the infected cells, which may account for the decreased expression levels of ARE-mediated antioxidant and phase-II enzymes [61]. The administration of the antioxidant drug N-acetyl cysteine (NAC) to *M. tuberculosis*-infected guinea pigs partially restored the serum total antioxidant capacity, with decreases in the lung and spleen bacterial counts and the severity of lesion necrosis. In summary, progressive oxidative stress during experimental TB in guinea pigs is partly caused by a defect in host antioxidant defenses, which can be partially restored by the administration of antioxidants. Rockwood et al. found that heme oxygenase 1 (HO-1) expression was markedly increased in rabbits, mice, and non-human primates during *M. tuberculosis* infection and decreased gradually during tuberculosis treatment. Interestingly, they also showed that ESAT-6-dependent stimulation of ROS results in HO-1 production by inducing nuclear translocation of NRF2 [62]. Many pathological effects observed in cells infected with *M. tuberculosis* are associated with bacterial production of the virulence factor ESAT-6, which is secreted into the host cell cytosol [63]. Actually, ESAT-6 is involved in the inhibition of phagolysosome fusion in *M. tuberculosis*-infected phagocytes [64]. It is thought that ESAT-6 disrupts phagosome membranes, thereby inhibiting phagolysosome fusion and allowing the bacillus to escape into the cytoplasm [65,66]. Since ESAT-6 is not restricted to *M. tuberculosis*, but is also present in some environmental strains including *M. kansasii*, *M. marinum*, and *M. szulgai* [67], these three other NTM strains may also induce ROS in the same way by ESAT-6.

HO-1, which is a cytoprotective enzyme, is directly modulated by Nrf2 [68]. There have been some reports on the relationship between HO-1 and *M. tuberculosis* infection [69,70,71]. Using freshly resected human lung tissue and HO-1-deficient mice, Chinta et al. showed that HO-1 in myeloid cells was important for the regulation of inflammation and free-radical-mediated tissue damage in TB [69]. In contrast, Costa et al. demonstrated that pharmacological inhibition of HO-1 rather suppressed *M. tuberculosis* infection in vivo by a mechanism dependent on T lymphocytes [70]. Moreover, Wu et al. showed that HO-1 polymorphism was associated with TB susceptibility in the Chinese Han population [71]. These reports indicate that HO-1 is associated with TB.

It has been reported that NRF2 plays an important role in TB infection. To elucidate the genes potentially regulated by NRF2 in TB, a meta-analysis of published gene expression datasets was conducted [72]. In this meta-analysis, an NRF2-mediated 17-gene signature (*GBP1*, *GCH1*, *HERC5*, *HLA−DMA*, *HLA−DMB*, *IFITM3*, *ISG20*, *MOV10*, *MT2A*, *OAS1*, *PARP9*, *PSMB9*, *RARRES3*, *SAMD9*, *SCARB2*, *STAT1*, *WDFY1*) was identified. These genes reflected a cluster of gene ontology terms highly related to TB physiology. They reported that the 17-gene signature can be used to distinguish TB patients from healthy controls and patients with latent TB infection, pneumonia, or lung cancer. Furthermore, the NRF2-mediated gene signature can be used as an indicator of the efficacy in anti-TB therapy. Thus, they confirmed the central role of NRF2 in TB pathogenesis [72]. In fact, to elucidate the role of NRF2 in tuberculous infection, experiments in which NRF2-deficient mice were infected with *M. tuberculosis* aerially were performed [73]. In this study, significant reductions in granuloma formation and tubercle bacilli in granulomas were noted in the NRF2-deficient mice 27 weeks after infection, concurrently with higher expressions of IL-2 and IL-13 mRNA. This finding suggests that NRF2 acts protectively against TB infection. In contrast, Rothchild et al. demonstrated that NRF2 drives expression of the cell-protective signature in alveolar macrophages and impairs the control of early bacterial growth 10 days after *M. tuberculosis* infection using NRF2-deficient mice [74].

Taken together, the role of NRF2 in the pathogenesis of *M. tuberculosis* infection appears to differ between the early and late stages of infection.

## 6. The Role of NRF2 in NTM Infection

There are not many reports regarding the role of NRF2 in NTM infection compared to those in TB infection. Nevertheless, Bonay et al. showed that human THP-1-derived macrophages infected with *M. abscessus* showed increased ROS production and cell necrosis, whereas *M. abscessus* infection triggered activation of the NRF2 signaling pathway. In addition, pretreatment of macrophages with sulforaphane (SFN), an activator of NRF2, followed by *M. abscessus* infection significantly decreased mycobacterial burden due to apoptosis [75,76]. Interestingly, this macrophage apoptosis was caspase 3/7-independent, but p38 MAPK-dependent. In this study, they showed that NRF2 stimulators may be interesting as future therapeutic options as a supplement to the standard multi-drug therapies used in pulmonary *M. abscessus* patients [75,76].

As for *M. avium* infection, the role of the oxidative stress sensor KEAP1 was reported. Awuh et al. proposed that KEAP1 negatively regulates inflammatory responses in *M. avium*-infected human primary macrophages [77]. Although this might be important to avoid excessive inflammation, they suggested that a negative effect could facilitate intracellular growth of *M. avium* [77].

There have been a few reports on the relationship between HO-1 and *M. avium* infection [78,79,80]. HO-1-deficient mice were used in all of these studies. Then, HO-1-deficient mice were more susceptible to *M. avium* infection than WT mice. In particular, Regev et al. reported that HO-1 promoted granuloma development and protected against the dissemination of *M. avium* [78]. These reports indicate that HO-1 is also associated with *M. avium* infection.

Recently, Nakajima et al. reported that NRF2-deficient mice were highly susceptible to *M. avium* bacteria compared to wild-type mice [81]. Surprisingly, NRF2 did not affect the level of oxidative stress or Th1 cytokine production in this *M. avium*-infection model. Comprehensive transcriptome analysis and subsequent ex vivo analysis showed that natural resistance-associated macrophage protein 1 (NRAMP1) and HO-1, regulated by NRF2, were essential in defending against *M. avium* infection due to the promotion of phagolysosome fusion and granuloma formation, respectively. In addition, treatment with SFN increased resistance to *M. avium* with increased lung expressions of NRAMP1 and HO-1 in wild-type mice. Since the *NRAMP1* gene is one of the important disease susceptibility genes of pulmonary NTM disease in humans [82], this NRF2-NRAMP1 pathway is fascinating in terms of elucidating the pathophysiology of pulmonary NTM infection. Thus, NRF2 was thought to be a critical determinant of host resistance to *M. avium* infection by a mechanism other than controlling oxidative stress. Consistent with reports of a wide variety of functions of NRF2, their study showed that NRF2 plays a role in the regulation of NRAMP1.

## 7. Aging and NRF2 in Mycobacterial Infection

Increased oxidative stress, which is a major feature of aging, has been implicated in a variety of age-related diseases. In aging, oxidative stress is increased, whereas antioxidant enzymes decrease, and the adaptive response to oxidative stress decreases. It has been reported that the ability of NRF2 to respond to oxidative stress decreases with aging [83]. Decreased antioxidant capacity due to a decrease in NRF2 functions might contribute to several age-related diseases.

In developing countries, TB rates are highest among the young, reflecting primary transmission in this age group. On the other hand, in the United States and other developed countries, the rate of TB among the elderly is higher than among the young and children, reflecting reactivation of the disease due to age-related decline in immunity [84].

As for the relationship between aging and NTM infection, a total of 1445 patients with treatment-naïve pulmonary NTM who were newly diagnosed between July 1997 and December 2013 were examined [85]. Upon multivariable analysis, the LASSO method demonstrated that old age (≥65 years); male sex; low body mass index (<18.5 kg·m^2^); underlying diseases including chronic pulmonary aspergillosis, malignancy, and chronic heart or chronic liver disease; and the erythrocyte sedimentation rate (ESR) were significantly associated with mortality in pulmonary NTM disease. This study showed that old age (≥65 years) is an independent factor associated with a poor prognosis in pulmonary NTM disease.

These epidemiological studies clearly demonstrated that mycobacterial infection is increasingly found in the elderly, but the underlying mechanisms are unclear. It has been reported that the expression of the *SOCS3* gene is increased and the expression of *Bcl2* gene is decreased with age, resulting in inhibition of Th1 cell differentiation [86]. A study in which aged and young mice were infected with NTM bacteria was also reported [80]. In this study, an attenuated HO-1 response with diffuse inflammation, a high burden of mycobacteria, poor granuloma formation, and decreased survival were observed in aged mice after infection, whereas young mice showed tight, well-defined granuloma, increased HO-1 expression, and increased survival. Taken together, higher susceptibility of the elderly to pulmonary NTM infection is partly caused by attenuated HO-1 responses, subsequent upregulation of *SOCS3*, and inhibition of *Bcl2*, leading to programmed cell death of macrophages and sustained infection. Since HO-1 is induced under the influence of NRF2 in young mice in response to NTM infection [81], it is likely that increased susceptibility to mycobacterial infection in the elderly is related to the decline of NRF2 functions with advancing age.

## 8. Developing New Therapeutic Options Targeting NRF2 and Oxidative Stress for NTM and TB Infection

Vilchèze et al. reported that vitamin C sterilizes in vitro cultures of *M. tuberculosis*, because vitamin C treatment increases ROS (superoxide, hydrogen peroxide, and hydroxyl radicals) production via the Harber–Weiss and Fenton reactions [87]. In *M. tuberculosis* cells, the production of these ROS leads to DNA damage and alteration of lipids and redox balance. In addition, they have shown that the combination of isoniazid/rifampicin with vitamin C sterilized the in vitro cultures of drug-susceptible *M. tuberculosis* more rapidly than antituberculous drugs alone [88]. These reports indicate that vitamin C acts to defend against TB infection. In fact, vitamin C deficiency has been linked to *M. tuberculosis* infection in the clinical setting [89]. A clinical trial showed the effectiveness of vitamin C supplementation for the treatment of TB, which merits further investigation [90].

**Figure 3:**
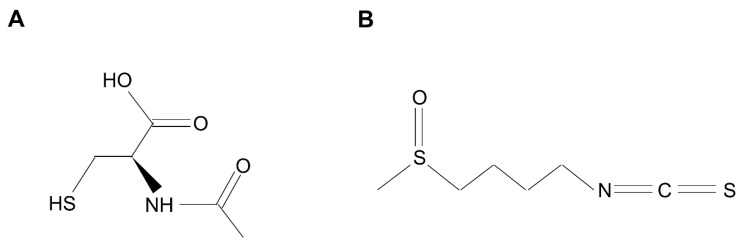
Chemical structures of N-acetyl cysteine (**A**) and sulforaphane (**B**).

NAC is widely used in patients with chronic lung diseases, including TB, because of its mucolytic and anti-oxidant activities [91,92,93,94]. The chemical structure of NAC is shown in Figure 3A. Pulmonary TB is associated with increased oxidative stress, increased lipid peroxidation, and decreased glutathione (GSH) levels. NAC effectively increases GSH levels, inhibits lipid peroxidation, and decreases ROS levels [91]. Furthermore, NAC could induce the expression of ARE-dependent enzymes via NRF2 activation [5,95], although the detailed mechanism was not elucidated. The usefulness of NAC as an adjuvant treatment agent for TB has been reported. Mahakalkar et al. showed that co-administration of NAC with a standard regimen, such as isoniazid + rifampicin + pyrazinamide + ethambutol for 2 months (2 HRZE) and isoniazid + rifampicin for 4 months (4 HR), to smear-positive patients with pulmonary TB brought about significantly faster sputum negativity, improved the radiological response, increased weight, raised serum GSH peroxidase (GPx) levels, and rectified the deregulated immune response [94]. Interestingly, Amaral et al. showed that NAC exerts direct antibiotic activity against mycobacteria other than through its antioxidant effects [96,97]. NADPH oxidase is a major source of ROS in eukaryotic cells [98]. To investigate whether NAC acts independently of NADPH-derived ROS inside macrophages, *gp91Phox*-deficient (gp91Phox^−/−^) mouse macrophages, which lack the major subunit of NADPH and have no NADPH activity, were infected with *M. tuberculosis*. Since treatment with NAC inhibited bacterial growth in both wild-type and gp91Phox^−/−^ macrophages, they suggested that the antimicrobial activity of NAC is not dependent on a fully functional host NADPH oxidase system. Shiozawa et al. reported that NAC-mediated protection against *M. avium* occurs through induction of an antibacterial peptide human β-defensin-2 (HBD-2) in A549 cells and murine β-defensin-3 in a mouse lung infection model [97]. NAC treatment alone reduced mycobacterial load in the lungs of *M. avium*-infected mice. Thus, the antimycobacterial effects of NAC may be due to not only its antioxidant effect, but also its direct antimicrobial effect. More clinical studies are needed to verify the adjuvant effect of NAC in patients with pulmonary TB and NTM diseases.

SFN is an organosulfur compound that belongs to the isothiocyanate group and is mainly found naturally in cruciferous vegetables, such as cauliflower, broccoli, and cabbage [99]. The chemical structure of SFN is shown in Figure 3B. It is considered to be a natural antioxidant, which is most abundantly present in broccoli sprouts, along with indole-3-carbinol and diindolylmethane [99]. SFN activates NRF2 through the modification of cysteines of KEAP1, resulting in the induction of the phase-II (carcinogen-detoxifying) enzyme in cells [100]. SFN is also known to have strong free radical scavenging properties and can bind to several oxidizing species, including superoxide, peroxide, and hydroxyl radicals, apart from being a powerful NRF2 pathway activator [101]. In addition, SFN has a broad spectrum of activity and has shown extraordinary potential as an antioxidant, antitumor, anti-angiogenic, and anti-inflammatory agent [99]. The antimicrobial activity of SFN against Gram-positive, as well as Gram-negative, bacterial strains, was tested. SFN in particular exhibited strong antimicrobial activity against *Helicobacter pylori* [102]. Patients with chronic obstructive pulmonary disease (COPD) showed inhibition of the NRF2 signaling pathway, which was associated with decreased phagocytosis of macrophages [103]. SFN-treated alveolar macrophages isolated from COPD patients were infected with *Haemophilus influenza* and *Pseudomonas aeruginosa*. These cells showed restored macrophage phagocytosis function and improved bacterial clearance due to the upregulation of the scavenger macrophage receptor with collagenous structure (MARCO) by NRF2 [103]. Although there are no reports regarding the efficacy of SFN in TB infection, the usefulness of SFN against *M. abscessus* and *M. avium* infections has been reported [75,81]. Thus, control of oxidative stress and activation of NRF2 might become novel therapeutic options for treatment of NTM and TB infections.

## 9. Conclusions

ROS and RNS are generated by phagocytic cells in response to invaded pathogens. The generation of these molecules is crucial for pathogen clearance in many infections. However, mycobacteria are resistant to ROS and survive in phagocytes because of their ability to produce antioxidant molecules. To kill mycobacteria, phagocytes amplify the generation of ROS by interaction with Th1 cells through the coactivation of IFNγ/IL12 pathways. During mycobacterial infection, ROS activates NRF2, a critical regulator of oxidative stress by inducing a battery of antioxidant molecules, in infected phagocytes. However, an animal study of *M. avium* infection showed that NRF2 did not affect the level of oxidative stress, but induced HO-1 and NRAMP1, which promote phagolysosome fusion and granuloma formation, respectively. Thus, NRF2 exerts antimycobacterial effects by a mechanism other than controlling oxidative stress. A schematic diagram of the role of NRF2 in *M. avium* infection is shown in Figure 2. NRF2 activation in response to oxidative stress decreases with aging. Mycobacterial infection is increasingly found in the elderly. In aged mice, higher susceptibility to NTM infection was observed with attenuated HO-1 responses. Since HO-1 is a downstream molecule of NRF2, increased susceptibility to mycobacterial infection in elderly persons may be related to decreased NRF2 functions with advancing age. The antimycobacterial effects of vitamin C, NAC, and SFN have been proposed. All of these drugs’ actions are related to the modification of cellular oxidative stress levels and activation of NRF2. Thus, control of oxidative stress and activation of NRF2 might become novel therapeutic options for treatment of mycobacterial infections in the near future.

In conclusion, the number of multidrug-resistant TB patients and pulmonary NTM patients is increasing. The role of NRF2 in mycobacterial infection has not been fully elucidated. Analyses of NRF2 knockout mice have shown that NRF2 acts both protectively and exacerbatively against TB infection. As for NTM infection, there is a report that NRF2 protects against *M. avium* infection by inducing HO-1 and NRAMP1, which contributes to granuloma formation and P-L fusion, respectively. These findings suggest that NRF2 plays an important role in the control of mycobacterial infection, although there are not many papers. Therefore, the controlling mechanisms should be elucidated at molecular and cellular levels to develop new treatment in the near future.
